# Paroxysmal abdominalgia as a non-motor wearing off phenomenon in Parkinson’s disease. A case series and literature review

**DOI:** 10.1016/j.prdoa.2025.100368

**Published:** 2025-07-16

**Authors:** Abdalmalik Bin Khunayfir, Stewart A. Factor

**Affiliations:** Jean & Paul Amos PD & Movement Disorders Program, Department of Neurology, Emory University School of Medicine, United States

**Keywords:** Parkinson’s disease, Non-motor symptoms, Nociplastic pain, Abdominalgia

## Abstract

**Background:**

Paroxysmal abdominalgia (PxA) is an underrecognized, debilitating form of abdominal pain that manifests during wearing-off (WO) periods in Parkinson’s disease (PD). Despite its profound impact, PxA remains poorly described, complicating diagnosis and management.

**Methods:**

We conducted a retrospective case series of patients with PD and recurrent abdominal pain linked to WO episodes. Demographic, clinical, and detailed pain data were extracted from records. Patients with alternative gastrointestinal (GI) causes were excluded. Data were analyzed descriptively and compared with existing literature.

**Results:**

Five patients (3 males, mean disease duration 14.4 years) met inclusion criteria. PxA was characterized by severe abdominal pain—described as twisting, squeezing, or tightness—that consistently occurred during WO states and frequently led to emergency department visits and repeated GI evaluations which were unremarkable. Symptoms were often associated with anxiety and panic attacks. Standard analgesics and GI therapies were largely ineffective. Extra carbidopa/levodopa doses provided variable relief; apomorphine bolus injections and continuous subcutaneous foslevodopa/foscarbidopa infusion appeared to offer significant benefit in eligible patients. PxA symptoms aligned best with the nociplastic category of the PD Pain Classification System.

**Conclusions:**

PxA is a severe, non-motor complication of PD that likely represents a form of nociplastic pain linked to dopaminergic fluctuations. Increased awareness is needed to reduce misdiagnosis and inappropriate interventions. Further research is required to elucidate underlying mechanisms and guide targeted therapy.

## 1 Introduction

Abdominal pain in Parkinson's disease (PD) patients can manifest in various forms, often complicating diagnosis, and management. The variability of abdominal pain includes episodes of cramping and bloating, abdominal wall stiffness and dystonia, and epigastric pain that may resemble ulcer pain or even an acute abdomen [1]. Rarely, abdominal pain can manifest as a severe and debilitating paroxysmal feature of wearing off (WO) phenomenon, which will be referred to here as *paroxysmal abdominalgia* (PxA). There are few such cases reported in the literature [[2], [3], [4]]. This type of pain can have variable presentations, localization, and severity, but they consistently occur during the WO episodes and are often quite debilitating and confusing to patients and physicians. Here, we describe a case series of five PD patients with PxA and provide literature review of the topic.

## 2 Case series (summarized in Table 1)

This retrospective record review was completed with a waiver of consent that was approved by the Emory University IRB. Patients were included if they had clinical diagnosis of PD and PxA. Demographic and clinical data, including PD duration, age of onset, Hoehn & Yahr (HY) stage, and medications were obtained from electronic medical records. Detailed characterizations of the abdominal pain (onset, duration, triggers, and associated symptoms), as well as therapeutic interventions were extracted. Records were screened for negative findings on gastrointestinal (GI) evaluations (e.g., imaging, endoscopy, colonoscopy) and patients were excluded if a clear alternative GI etiology (e.g., severe constipation) was found. Data were analyzed descriptively to identify patterns, common features, and potential treatment responses, and were compared with the limited existing literature.

### 2.1 Case 1

An 84-year-old man with a 22-year history of PD (HY stage 4) had been experiencing episodes of abdominal pain and tightness for the prior four years. At the onset of the pain, the patient would begin pacing the floors due to a sense of restlessness. He sometimes had his wife push on his abdomen while seated on the toilet to help relief the pain. These episodes, which could last hours and happen only during WO periods, were accompanied by anxiety and sometimes triggered by eating or exertion. Despite seeing multiple gastroenterologists, and undergoing an upper endoscopy, no formal GI diagnosis was made. He was treated with multiple pain medications, but none provided significant relief (Table 1). His medication regimen included carbidopa/levodopa (C/L) 25/100 mg, 3 tablets five times a day, and he had found that taking an extra dose sometimes alleviated his symptoms. Subcutaneous apomorphine hydrochloride bolus injections ultimately resulted in an 80 % improvement in abdominal pain.

**Table 1:** Demographics and clinical features of PxA in our cohort.

**Details**	**Patient 1**	**Patient 2**	**Patient 3**	**Patient 4**	**Patient 5**
**Age and Gender**	84 M	69F	75 M	53 M	75F
**Age at time of PD Diagnosis**	62	58	63	42	51
**Hoehn & Yahr**	Stage 4	Stage 2	Stage 2	Stage 2	Stage 4
**Main PD Complaints at PxA onset**	Gait imbalance, motor fluctuations, dyskinesia	Motor fluctuations	Motor fluctuations, dyskinesia	Gait imbalance, motor fluctuations, dyskinesia	Motor fluctuations, dyskinesia
**Age at time of paroxysmal abdominalgia onset**	80	67	74	51	60
**Description of paroxysmal abdominalgia**	Abdominal “lump or knot”, pain, tightness, lasting hours	Squeezing and twisting pain, radiating from umbilicus to chest or throat, lasting hours	Abdominal pain, bloating, lasting hours	Periumbilical pain, bloating.	Midsternal /epigastric pain radiating to throat. Lasting hours.
**Triggers**	Eating. Exertion	Movement	−	−	Eating
**Related Symptoms during off periods**	Anxiety	Panic attacks	Panic attacks. Fear of eating.	Rigidity	Panic attacks, anxiety, sensation of “throat closing”. Leg pain
**Failed Medications**	Lorazepam, clonazepam, alprazolam, quetiapine, mirtazapine	Sertraline, mirtazapine, various C/L formulations Apomorphine hydrochloride injections – severe nausea	Entacapone, various C/L formulations (including levodopa inhalation powder)	Probiotics	Acetaminophen, oxycodone, pregabalin, buspirone, quetiapine, alprazolam, multiple C/L adjustments, duloxetine, aripiprazole, olanzapine
**Treatment and Outcome**	Temporary relief: venlafaxine Some relief: extra dose of C/L Significant relief: apomorphine hydrochloride injections	Some relief: extra dose of C/L Significant relief: Continuous 24 h/day subcutaneous infusion of foslevodopa/foscarbidopa	Some relief: Baclofen	Significant relief: extra dose of C/L during WO periods and C/L extended release at bedtime to help with morning symptoms.	Temporary relief: extra dose of C/L

Abbreviations: Carbidopa/levodopa, C/L; Paroxysmal Abdominalgia, PxA.

### 2.2 Case 2

A 69-year-old woman with PD (HY stage II), diagnosed 11 years earlier, presented for evaluation of her motor fluctuations. She had been taking C/L 25/100, 2 tablets every 3 h. Around the ninth year of her disease, she began experiencing abdominal pain that radiated from her umbilicus to the epigastric area, often radiating to the chest or throat. She described the sensation as “squeezing and twisting.” These episodes occurred during WO periods and could last for hours. The pain sometimes was aggravated by moving. These episodes were accompanied by panic attacks, where she feels she is about to die. Multiple visits to the emergency room (ER) for the pain showed no clear etiology apart from a diagnosis of “constipation”, which has been intensively treated with no significant relief. Despite extensive evaluation by two gastroenterologists, including CT scan of the abdomen, colonoscopy, and upper endoscopy, no underlying cause was identified. The patient had mild relief of the pain if she pushed her belly against marble ledge in her bathroom or lie down on the bed with a pillow across her abdomen. Various formulations of oral C/L and other medications were trialed for pain, but none provided meaningful relief (Table 1). Subcutaneous apomorphine hydrochloride bolus injections were tried but was not tolerated due to severe nausea. With the addition of the recently approved continuous 24 h/day subcutaneous infusion of foslevodopa/foscarbidopa, PxA has stopped happening after the patient was on the infusion for a month.

### 2.3 Case 3

A 75-year-old man with PD (HY stage II) for the past 12 years presented with significant motor fluctuations, with over 3 h of severe off times daily. He was on a regimen of C/L 25/100 mg, 2 tablets, 7–8 times a day. By the 11th year of his disease, the patient began experiencing abdominal pain and bloating, which made eating difficult and led to a 40-pound weight loss. He developed dyspnea associated with the pain and presented to the ER, where a CT scan revealed a large stool burden and an abnormal positioning of the sigmoid colon in the upper central abdomen, raising concern for sigmoid volvulus. Although initially prepared for surgical intervention, his condition improved significantly with intensive treatment for constipation, negating the need for surgery. However, abdominal pain episodes continued to occur. Overtime, it became clear that they occurred during his WO periods. Additionally, during these episodes, he experienced fear of eating and panic attacks, characterized an overwhelming feeling that he is going to die due to breathlessness. Work up by gastroenterology and general surgery for the abdominal pain and the weight loss included CT scans and endoscopy, all of which were negative. Baclofen provided minor relief, whereas other medications were ineffective (Table 1).

### 2.4 Case 4

A 53-year-old man with an 11-year history of PD (HY stage II) presented with motor fluctuations over the prior two years. He was taking one tablet of C/L 25/100 mg every two hours, up to seven times daily. Associated with his WO times, he began experiencing periumbilical abdominal pain and bloating without clear triggers. Because he has a family history of colon cancer, he had a colonoscopy, which was negative, and later a repeat colonoscopy due to continued episodes of abdominal pain, which was also negative. He eventually realized that the abdominal pain consistently signaled the start of his WO episode. He experienced significant relief after taking extra dose of C/L during WO periods and C/L extended release at bedtime to help with morning symptoms (Table 1).

### 2.5 Case 5

A 75-year-old woman diagnosed with PD at age of 51 developed severe motor fluctuations and dyskinesia. She was taking C/L 25/100 mg every 1.5 h. She later developed severe pain that “goes through the abdomen and the esophagus”. Because eating sometimes provoked the pain, she abstained from eating, leading to notable weight loss. It was later noticed that the pain mainly occurred during WO. To address her motor fluctuations, she underwent bilateral GPi DBS, with significant improvement of her dyskinesia and motor off features, but not the pain. Extensive diagnostic evaluations by gastroenterology and cardiology, including upper endoscopy and colonoscopy, have found no cause for the pain. Although C/L initially provided some relief, its efficacy diminished over time, and various other medications proved ineffective (Table 1).

## 3 Discussion

Our findings highlight PxA as an underrecognized non-motor manifestation of WO in PD, posing diagnostic and therapeutic challenges. Pain is a complex non-motor feature of PD and is estimated to occur in ∼ 40 % of the patients [5]. Although various forms of pain are documented, PxA has received limited attention in the literature. PxA is defined as abdominal pain, that is typically severe, and occurs during the WO of periods in PD. Five cases of PxA are documented in the literature [[2], [3], [4]], with clinical findings summarized in supplemental table 1. In our cohort (summarized in Table 1), a total of five patients were identified with PD and PxA, all of whom had long-standing PD with Hoehn & Yahr stages ranging from 2 to 4. PxA onset occurred 9–18 years post-PD diagnosis, emerging after onset of motor fluctuations. As PxA is linked to WO periods, it tended to happen early morning and before taking the scheduled doses of C/L. Pain descriptions varied—abdominal tightness, squeezing, twisting, discomfort, and bloating—and episodes were severe and typically lasted for hours. All patients were seen by gastroenterology, one patient by cardiology, and two had multiple ED visits. Diagnosis of PxA is significantly delayed because it is not recognized as a manifestation of PD and/or labeled as psychogenic. All patients had at least one endoscopy (either upper, lower, or both); one patient had more than one. Two patients had notable weight loss, as eating tended to make the pain worse. Two patients identified movement or physical exertion as triggers; however, the underlying mechanism for this association remains unclear. Panic attacks and anxiety were present in four out of five patients, whereas only one prior case reviewed in literature had similar symptoms (supplemental table 1, and Table 1) [2]. Various medications, such as analgesics, antispasmodics, antidepressants, and benzodiazepines, were ineffective, offered limited or no sustained benefit. Extra doses of C/L can provide mild to significant relief, likely depending on how advanced the disease is. The most significant relief came from the rescue medication apomorphine hydrochloride injections (patient 1), which has been reported in one previous patient (supplemental table 1) [2] and from continuous subcutaneous infusion of foslevodopa/foscarbidopa (patient 2). Perhaps other rescue drugs such as inhaled levodopa and continuous infusions such as jejeunal levodopa infusions or subcutaneous apomorphine infusion could provide similar benefit; however, supporting data are currently lacking. One of our patients underwent bilateral GPi DBS, which did not improve PxA. Although STN DBS has been reported to help PxA in one patient [4], none of our patients received this treatment.

Given the limited prior characterization of PxA, its integration into existing frameworks of PD-related pain remains challenging. The most widely cited classification system was originally proposed by Ford in 1998 and subsequently refined in 2010, delineating five categories of PD-related pain: musculoskeletal, dystonia-related, akathisia, neuropathic/radicular, and central pain [5,6]. More recently, the Parkinson’s Disease Pain Classification System (PD-PCS), introduced in 2021, offers a mechanistic taxonomy categorizing PD-related pain as nociceptive, neuropathic, or nociplastic (supplemental table 2 [[7], [8], [9]]). Among these, PxA appears most consistent with the nociplastic category. This is because nociplastic pain is linked to dopaminergic fluctuations (i.e., worsening during wearing-off), tends to be poorly localized, and is frequently associated with psychiatric and behavioral symptoms. Such pain results from altered nociceptive processing without an identifiable peripheral or central structural cause. It is important to note that before labeling pain as nociplastic, other types must be excluded — for example, ensuring that visceral abdominal pain is not due to constipation-related nociceptive pain or dermatomal patterns suggestive of neuropathic pain. Of note, some patients in our study had some triggers (such as worsening of symptoms with movement or eating), but this Nonetheless, the current study is limited by a small sample size (n = 5) and its single-center design. Further research is necessary to clarify the mechanisms of PxA and develop more effective treatments.

## Financial disclosures

Financial disclosures for the Previous 12 Months: Dr. Bin Khunayfir has nothing to disclose. Dr. Factor has the following disclosures: Honoraria: Biogen, Takeda, Neurocrine. Grants: Sun Pharmaceuticals Advanced Research Company, Aspen, Biohaven, Neurocrine, Rho inc, CHDI Foundation, Michael J. Fox Foundation, NIH 1 P50 NS123103-01, NIH 1R01NS125294-01, Parkinson Foundation. Royalties: Demos, Blackwell Futura, Springer for textbooks, Uptodate. Other Signant Health.

## Ethical compliance statement

This retrospective record review was completed with a waiver of consent that was approved by the Emory University IRB (IRB00002451, Approval May 21, 2018). No identifiable patient information is included in this work. We confirm that we have read the Journal’s position on ethical publication and affirm that this work is consistent with those guidelines.

## Funding sources

No specific funding was received for this work

## CRediT authorship contribution statement

**Abdalmalik Bin Khunayfir:** Writing – original draft, Investigation, Data curation. **Stewart A. Factor:** Writing – review & editing, Supervision, Conceptualization.

## Declaration of competing interest

The authors declare that they have no known competing financial interests or personal relationships that could have appeared to influence the work reported in this paper.
