# Supplementary material for: Paroxysmal abdominalgia as a non-motor wearing off phenomenon in Parkinson’s disease. A case series and literature review
Source: Clin Park Relat Disord. 2025 Jul 16;13:100368. doi: 10.1016/j.prdoa.2025.100368 (PMC12291550; doi:10.1016/j.prdoa.2025.100368)
Supplement: Supplementary Data 1 [file mmc1.docx]

**Supplemental tables for manuscript “Paroxysmal abdominalgia as a non-motor wearing off phenomenon in Parkinson’s disease. A case series and literature review”**

**Supplemental table 1:** Demographics and clinical features of PxA in the literature.

| **Details** | **Case 1 (Factor SA et al.)** | **Case 2 (Rana AQ et al.)** | **Case 3 (Rana AQ et al.)** | **Case 4 (Kataoka H et al.)** | **Case 5 (Kataoka H et al.)** |
| --- | --- | --- | --- | --- | --- |
| **Age and Gender** | 75 F | 61 M | 71 M | 67 M | 74 F |
| **Age at time of PD Diagnosis** | 57 | 58 | 67 | 63 | 63 |
| **Hoehn & Yahr** | 2* | 2* | 2* | 2 | 3 |
| **Main PD Complaints at PxA onset** | Motor fluctuations, akinetic rigid, dyskinesia. | Motor fluctuations | Motor fluctuations | Motor fluctuations, freezing of gait | Motor fluctuations |
| **Age at time of paroxysmal abdominalgia onset** | 63 | 61 | 70 | 63* | 72 |
| **Description of paroxysmal abdominalgia** | Pain localized to left side of diaphragm, progressing from fluttering to severe sharp pain, lasting 3-4 hours, worst in mornings and evenings | Severe abdominal pain in forms of cramps | Abdominal pain in early morning | Evening daily pain, in lower and upper abdomen, feeling of a muscle contraction or cramp. Palpable abdominal muscle contraction. | Persistent or recurrent, daily, intolerable, lower abdominal pain, feeling of muscle contraction, cramp and squeezing. |
| **Related Symptoms during off periods** | Anxiety and panic attacks | --- | --- | --- | --- |
| **Failed Medications** | Clonazepam, alprazolam, amitriptyline, lithium, various dopamine agonists, and pain medications (hydrocodone, codeine, acetaminophen, morphine, and fentanyl). | Analgesics and spasmolytics | --- | Ropinirole, zonisamide | Rotigotine, pregabalin, valproic acid, duloxetine, rotigotine patch, and imipramine. |
| **Treatment and Outcome** | Subcutaneous apomorphine injections produced significant pain relief | Addition of controlled-release C/L reduced pain recurrence | Early morning pain relieved only by next dose; improved with bedtime controlled-release C/L | Short term benefit: increase in C/L dose, apomorphine injection, etizolam,  Tramadol, lidocaine infusion to rectus muscles of the abdomen | Short term benefit: increase in C/L dose. Pain completely resolved with deep brain stimulation of the subthalamic nucleus. |

* The information regarding the Hoehn and Yahr stage and the age at the onset of paroxysmal abdominalgia was not explicitly stated in the referenced article. Therefore, the values presented here are inferred based on the details provided in the case descriptions.

Abbreviations: Carbidopa/levodopa, C/L; Paroxysmal Abdominalgia, PxA

**Supplemental table 2:** Pain classification using Parkinson’s Disease Pain Classification System (PD-PCS). PxA is introduced as part of the nociplastic pain.

| **Pain Type** | **Definition** | **Examples** | **Relation to dopaminergic fluctuations** | **Comments** |
| --- | --- | --- | --- | --- |
| **Nociceptive Pain** | Pain arising from activation of peripheral nociceptors without damage to the somatosensory system. | Rigidity. Musculoskeletal pain (e.g., osteoarthritis). Dystonic spasms. Visceral pain (e.g., constipation). Suboccipital and paracervical neck pain (i.e. coat hanger pain) due to orthostatic hypotension. | Tend to occur in both ON and OFF states. | Tend to affect trunk and back. |
| **Neuropathic Pain** | Pain due to a lesion of the somatosensory nervous system | Radiculopathy. Nerve Lesions. Polyneuropathy | Tend to occur in both ON and OFF states. | Affect extremities more often. |
| **Nociplastic Pain** | Pain without any evidence of somatosensory system lesion or peripheral nociceptive activation; due to altered central pain processing | Non-specific, fluctuating, and migratory pain. Restless legs syndrome. Akathisia. Non-motor off-state pain (e.g. PxA). | Worsen during OFF periods. | Poor localization. Related to dopaminergic fluctuations and associated with non-motor cognitive and behavioral symptoms (e.g., anxiety, restlessness, sweating, or psychomotor agitation) |

Abbreviations: Paroxysmal Abdominalgia, PxA
